# Elucidating factors contributing to disparities in pain-related experiences among adults with or at risk for knee osteoarthritis

**DOI:** 10.3389/fpain.2023.1058476

**Published:** 2023-02-22

**Authors:** Angela M. Mickle, Lisa H. Domenico, Jared J. Tanner, Ellen L. Terry, Josue Cardoso, Toni L. Glover, Staja Booker, Adriana Addison, Cesar E. Gonzalez, Cynthia S. Garvan, David Redden, Roland Staud, Burel R. Goodin, Roger B. Fillingim, Kimberly T. Sibille

**Affiliations:** ^1^ College of Medicine, Department of Physical Medicine and Rehabilitation, University of Florida, Gainesville, FL, United States; ^2^ College of Dentistry, Pain Research and Intervention Center of Excellence (PRICE), University of Florida, Gainesville, FL, United States; ^3^ College of Nursing, Department of Biobehavioral Nursing Science, University of Florida, Gainesville, FL, United States; ^4^ College of Public Health and Health Professionals, Department of Clinical and Health Psychology, University of Florida, Gainesville, FL, United States; ^5^ School of Nursing, Oakland University, Rochester, MI, United States; ^6^ Department of Psychology, College of Arts and Science, University of Birmingham Alabama, Birmingham, AL, United States; ^7^ College of Medicine, Department of Anesthesiology, University of Florida, Gainesville, FL, United States; ^8^ Department of Biostatistics, School of Public Health, University of Birmingham Alabama, Birmingham, AL, United States; ^9^ College of Medicine, Department of Rheumatology, University of Florida, Gainesville, FL, United States

**Keywords:** health disparities, clinical pain, physical function, experimental pain, social determinants of health, knee osteoarthritis

## Abstract

**Background and purpose:**

We and others have reported ethnic/race group differences in clinical pain, physical function, and experimental pain sensitivity. However, recent research indicates that with consideration for socioenvironmental factors, ethnicity/race differences become less or non-significant. Understanding of factors contributing to pain inequities are needed. Guided by the *NIA* and *NIMHD Health Disparities Research Frameworks*, we evaluate the contributions of environmental and behavioral factors on previously reported ethnic/race group differences in: (1) clinical pain, (2) physical function, and (3) experimental pain in individuals with knee pain.

**Methods:**

Baseline data from Understanding of Pain and Limitations in Osteoarthritis Disease (UPLOAD) and UPLOAD-2 studies were analyzed. Participants were adults 45 to 85 years old who self-reported as non-Hispanic white (NHW) or black (NHB) with knee pain. A health assessment and quantitative sensory testing were completed. Sociodemographics, environmental, health, clinical and experimental pain, and physical functioning measures were included in nested regressions.

**Results:**

Pooled data from 468 individuals, 57 ± 8 years of age, 63% women, and 53% NHB adults. As NHB adults were younger and reported greater socioenvironmental risk than the NHW adults, the term sociodemographic groups is used. With inclusion of recognized environmental and behavioral variables, sociodemographic groups remained a significant predictor accounting for <5% of the variance in clinical pain and physical function and <10% of variance in experimental pain.

**Conclusion:**

The incorporation of environmental and behavioral factors reduced relationships between sociodemographic groups and pain-related outcomes. Pain sites, BMI, and income were significant predictors across multiple models. The current study adds to a body of research on the complex array of factors contributing to disparities in pain-related outcomes.

## 1. Introduction

It is currently estimated that over 116 million individuals within the United States live with chronic pain making it a leading cause of disability, loss of work productivity, and loss of participation in life activities (1, 2). It has been reported that under-represented ethnic/race groups are disproportionately affected by both the prevalence and deleterious impact of chronic pain conditions (3, 4). A plethora of research over the previous twenty years indicates that non-Hispanic black (NHB) individuals along with other ethnic/race groups experience greater clinical pain, functional limitations, and experimental pain compared to non-Hispanic white (NHW) individuals (5–11). However, recent studies suggest socioenvironmental factors may account for some of the previously reported ethnic/race group differences. As many observational studies have not been able to match study participants on relevant socioenvironmental factors, imbalances in the representation by different ethnic/race groups likely contributed to observed disparities (12).

Health outcomes are greatly influenced by environmental and sociocultural factors (13–18). Several initiatives are underway to better understand factors contributing to health disparities and to design interventions to improve outcomes. *Healthy People 2030* objectives target social determinants of health to reduce disparities and improve overall health (19). Another effort in the pain research community is *Antiracism in Pain* (12, 20, 21). Additionally, two research frameworks are available. The NIA Health Disparities Research Framework was developed to improve the evaluation of and identify needs in aging and disparity research (22). The National Institute on Minority Health and Health Disparities Framework expanded on the NIA Framework by incorporating concepts from the socioecological model (23). The NIA Framework provides four levels of analysis and lists associated factors by topic and subtopic that are highly useful when trying to identify where a factor or measure aligns. The NIMHD Framework includes levels of influence and domains of influence which encompass and expand on the NIA Framework.

Our studies, Understanding of Pain and Limitations in Osteoarthritis Disease (UPLOAD) and UPLOAD-2, were designed to better understand the array of biopsychosocial factors contributing to ethnic/racial differences in pain-related outcomes in mid to older adults with knee pain with or at risk for knee osteoarthritis (OA). Across both studies, we have reported significant ethnic/race group differences in clinical pain and physical function (24–29) and experimental pain (24, 30–33). We have also shown ethnic/race group differences in factors associated with chronic pain including discrimination, pain catastrophizing, perceived stress, resilience, evening cortisol, nutritional supplement status, and sociodemographic factors (25, 26, 30, 34–42).

Aligning with the national initiatives underway, the purpose of our study is to investigate relationships between pain measures and environmental and behavioral factors as identified in the NIA and the NIMHD Health Disparities Research Frameworks. Working with combined and unduplicated data from the UPLOAD and UPLOAD-2 studies, the goal is to better understand the contribution of environmental and behavioral factors on the previously reported ethnic/race group differences in pain related measures. We aim to determine the contributions of environmental and behavioral factors specific to: (1) clinical pain, (2) physical function, and (3) experimental pain in mid to older adults with knee pain with or at risk for osteoarthritis.

## 2. Methods

### 2.1. Participants

Baseline data from two studies, UPLOAD and UPLOAD-2, were combined. Both are multi-site studies conducted at the University of Florida and the University of Alabama at Birmingham. Cross-sectional in design, the UPLOAD study was completed between January 2010 and October 2013 (*n* = 280). UPLOAD-2 is a prospective cohort study, which began in August 2015. Inclusion and exclusion criteria for both studies are described in detail (24, 34). The inclusion/exclusion criteria was the same for both studies. Participants were included if they were between the ages of 45 and 85 years and had knee pain for at least one month prior to screening. Participants were excluded from the primary studies if they: (1) had cognitive impairment; (2) used opioids on a daily basis; (3) were hospitalized for a psychiatric illness in the preceding year; (4) had a history of acute myocardial infarction, heart failure or uncontrolled hypertension (BP > 150/95 mm Hg); (5) had prosthetic knee replacements or other clinically significant surgery to the affected knee; (6) had peripheral neuropathy; and/or (7) had systemic diseases including rheumatoid arthritis, systemic lupus erythematosus or fibromyalgia. Pooled data included the cross-sectional baseline time points only combining identical measures. For both studies, participants were non-Hispanic black (NHB) and non-Hispanic white (NHW) community-dwelling adults between 45 and 85 years of age with knee pain consistent with or at risk for knee osteoarthritis (OA) as previously reported (24, 34). The University of Florida Institutional Review Board and the University of Alabama at Birmingham Institutional Review Board approved the studies and all participants provided informed consent. This manuscript follows the STROBE guidelines (43).

### 2.2. Procedures

Descriptions are limited to the procedures and measures relevant to address the study questions. Participants completed a baseline health assessment and a quantitative sensory testing session approximately one week apart. All participants who met inclusion/exclusion criteria who reported knee pain and completed the baseline health assessment were included. Participant characteristics of those screened but did not complete the baseline assessment were not collected. The measures below were selected from those available in both studies and align with the environmental and behavioral levels of analysis identified in the NIA and NIMHD Health Disparities Research Frameworks (22, 23). The studies did not include a sufficient number of measures to address sociocultural and biological levels of analysis and are not included in this investigation. Both studies used self-report measures to better understand the individual experiences of pain, function/disability and biobehavioral factors.

### 2.3. Measures

#### 2.3.1. Baseline characteristics

Data included age, sex, ethnicity/race, and current comorbidities. Per National Institutes of Health guidelines, individuals self-reported their ethnicity/race. Comorbidities were identified from the following list of conditions: high blood pressure, heart disease, cancer, diabetes, asthma/breathing problems, kidney disease, thyroid problem, stroke, seizure, chronic pain, neurological disorder, depression, other mental health condition, or other health problem. Height and weight measurements were taken, and body mass index (BMI) calculated.

#### 2.3.2. Environmental

##### 2.3.2.1. Individual

Education was combined from six categories into three categories (1 = high school or less, 2 = some college/bachelor's degree, 3 = graduate degree). Employment status data was combined from seven categories into three categories (0 = not working, temporarily laid off, 1 = student, disabled, other, 2 = working, retired). Income was collected based on the following financial categories (1 = $0–$9,999, 2 = $10,000–$19,999, 3 = $20,000–$29,999, 4 = $30,000–$39,999, 5 = $40,000–$49,999, 6 = $50,000–$59,999, 7 = $60,000–$79,999, 8 = $80,000–$99,999, 9 = $100,000–$149,999, 10 = $150,000 or higher). Current health insurance status was dichotomous (0 = no, 1 = yes).

##### 2.3.2.2. Community/ecological

The Area Deprivation Index (ADI) is a validated measure of objective socioeconomic status using 17 indicators such as housing, income, employment and education (44). The nine-digit zip code for each participant was used to identify the state (*n* = 466) and national (*n* = 466) level ADI. The Center for Disease Control Social Vulnerability Index (SVI) is another validated objective socioeconomic status using 15 indicators such as race/ethnicity, housing, employment and language (45). The census tract ID and county for each participant were used to assign the county SVI (*n* = 465) and census SVI (*n* = 422). A total of 44 participants provided a PO Box which was used as a proxy for SVI county. Two participants did not provide an address. Higher values on either index represent a worse social disadvantage. Due to the high correlation between ADI variables, spearman correlation was completed to select one variable. After correlation analysis, ADI national was most correlated with the outcomes of interest and included in the later analysis. Due to the homogeneity of participants living in the same county, the census SVI was used in the analysis rather than county SVI.

#### 2.3.3. Behavioral

##### 2.3.3.1. Perceived stress scale (PSS)

The PSS is a 14-item self-report questionnaire that measures perceived stress over the previous month using a five-point Likert scale from 0 = “Never” to 4 = “Very Often” (46). Total scores range from 0 to 40 with higher scores indicating greater perceived stress (*n* = 457). The PSS has been shown to have good internal consistency in previous research (*α *= 0.84–0.86) (46) with good consistency in our sample (*α *= 0.85).

##### 2.3.3.2. Smoking status

Participants were asked if they smoked at least 100 cigarettes in their lifetime. If yes, whether they were current smokers or had quit (0 = never smoked, 1 = previous smoker, 2 = current smoker) (*n* = 463).

##### 2.3.3.3. Life orientation test-revised (LOT-R)

The LOT-R is a 10-item self-report questionnaire that is a measure of optimism vs. pessimism (47). Items are rated on a 5-point Likert scale where 0 = “I disagree a lot” to 4 = “I agree a lot”. Scores range from 0 to 24 with higher scores indicating greater optimism (*n* = 462). The LOT-R has shown to have good internal consistency in previous research (*α* = 0.75) (48) and good consistency in our sample (*α *= 0.73).

##### 2.3.3.4. Experience of discrimination (EOD)

The EOD is an 18-item self-report questionnaire that measures how often people feel they have experienced unfairness on the basis of race, ethnicity, gender, age, religion, physical appearance, sexual orientation, or other characteristics (49, 50). It contains nine settings of “yes” or “no” whether discrimination occurred and the number of times. Settings include school, job seeking, at work, seeking housing, getting medical care, in a store or restaurant, getting credit, on the street or public setting, with the police or in the courts. The other nine items ask on a day to day how often observed indicators occurred ranging from never, once, two or three times, or four or more times. Scores range from 10 to 60 with higher scores indicating greater lifetime discrimination (*n* = 459). The EOD has shown to have good internal consistency (*α *= 0.74 or greater), and test-re-test reliability coefficients (*α* = 0.70) in previous research and good consistency in our sample (*α* = 0.76)(50).

##### 2.3.3.5. The multidimensional scale of perceived social support (MSPSS)

The MSPSS is a 12-item self-report measure that assesses an individual's perceived level of social support with family, friends, and significant others (51). Statements are rated on a 7-point Likert scale from 1 = “very strongly disagree” to 7 = “very strongly agree”. Scores range from 0 to 72 with higher scores indicating greater perceived level of support (*n* = 452). The MSPSS is one of the most extensively used social support measure and has shown high internal consistency (*α* = 0.88), and stability after three months (*α* = 0.85) in previous research and excellent consistency in our sample (*α* = 0.96) (52).

#### 2.3.4. Clinical pain and physical function

##### 2.3.4.1. Graded chronic pain scale (GCPS)

The GCPS is a self-report measure that assesses the severity of knee pain and the impact on daily activities over a 6-month period (53). The measure is comprised of two subscales. The characteristic pain intensity (CPI) (0–100 score) is calculated as the mean rating for current, worst and average pain from 0 to 10 where 0 = “no pain” and 10 = “pain as bad as it could be”. The disability score (0–100 score) is calculated as the mean rating for difficulty performing daily, social and work activities from 0 to 10 where 0 = “no change” to 10 = “extreme change”. Greater scores indicate higher pain intensity and greater physical disability (*n* = 466). The GCPS has demonstrated good internal consistency in previous research (*α* = 0.74) and the current sample (*α* = 0.84) (53).

##### 2.3.4.2. Western Ontario and McMaster universities osteoarthritis index (WOMAC)

The WOMAC is a validated measure of clinical knee pain and functioning over the previous 48-hours on three sub-scales including pain (0–20 score), stiffness (0–8 score), and function (0–68) (54). Sub-scores are summed for a total score ranging from 0 to 96 with higher scores indicating worse physical pain, stiffness, and functioning. For this study, pain (*n* = 467) and function (*n* = 468) subscales were used. The WOMAC has demonstrated good internal consistency (*α* = 0.84–0.95) across several previous studies (55–57) and in the current sample (*α* = 0.91).

##### 2.3.4.3. Pain sites

Participants were asked if they had pain on more days than not over the past three months based on bilateral areas including hands, arms, shoulders, neck, head/face, chest, stomach, upper back, lower back, knees, legs (other than knees), and/or feet/ankles (*n* = 468) (0–24 sites). Pain sites served as a covariate for pain severity in the model (58). Increasing number of pain sites has been linked to worse health outcomes and three or more pain sites is considered widespread pain (59, 60).

#### 2.3.5. Experimental pain

##### 2.3.5.1. Punctate temporal summation (hand and knee)

Punctate testing was conducted on the index knee and the back of the hand using a nylon monofilament calibrated to deliver a force of 300 grams (31). Participants were asked to provide a verbal pain rating of 0–100, where 0 = “no pain” and 100 = “the worst pain imaginable”, after a single contact of the monofilament. Then the participants were asked to provide a pain rating after a series of ten contacts delivered approximately one per second. The procedure was repeated twice at each location. Temporal summation (TS) was determined by subtracting the average pain rating of the series of ten contacts for each site minus the average of the single contact (*n* = 436).

## 3. Statistical methods

Data were checked for missingness, normality, and outliers. Chi-squared, Wilcoxon rank sum tests, or Spearman correlational tests were performed for all bivariate analyses between clinical pain (GCPS pain intensity, WOMAC pain), physical function (GCPS disability, WOMAC physical function), experimental pain (punctate TS) and environmental (education, employment, income, insurance, ADI, SVI) and behavioral (PSS, smoking, LOT-R, EOD, MSPSS) variables. Environmental and behavioral variables, except for health insurance, were significantly correlated with the outcome variables and were retained in the adjusted models. Multi-collinearity exclusion was set at 0.7. ADI National and SVI Census were associated at rho = 0.66 and retained.

Nested Linear Regression models were built with 3 sets of variables. Set 1 included environmental and behavioral variables including income, employment status, education level, ADI National, SVI Census, smoking history, LOT-R, EOD, MSPSS and PSS. Set 2 included the same measures with the additional explanatory variables including age, sex, income, number of pain sites, total comorbidities, and BMI. Finally, set 3 included all variables from set 2 and indicator variables for sociodemographic groups, NHB adults with low sociodemographic resources and NHW adults with high sociodemographic resources. All statistics were completed using SAS v9.4 (SAS Institute, Cary, North Carolina, United States). All tests were considered statistically significant at a 0.05 level of significance.

## 4. Results

### 4.1. Study sample

The sample included 468 individuals, 57.3 ± 7.7 years of age, 63.0% women, and 52.8% NHB participants. NHB participants were younger, had a higher BMI, reported significantly lower income, education, and employment status compared to NHW participants. Due to the significant sociodemographic differences between the NHB and NHW participants, only a subgroup for each are represented, ethnic/race group interpretations would be inaccurate. Therefore, we use the term *sociodemographic group* when reporting findings. Descriptive information is provided in Table 1.

**Table 1:** Study sample.

Variable	Total (*N* = 468)	NHB (*n* = 247)	NHW (*n* = 221)	*p*-value
**Environmental and Behavioral Measures**				
**Income, *N* (%)**				**<0** **.** **0001**
<$50,000	325 (69.4)	206 (83.4)	119 (53.8)	
>$50,001	136 (29.1)	37 (15.0)	99 (44.8)	
Not reported	7 (1.5)	4 (1.6)	3 (1.4)	
**Employment, *N* (%)**	** **	** **	** **	**<0** **.** **0001**
Not Working	69 (14.8)	50 (20.2)	19 (8.6)	
Student/other	89 (19.1)	61 (24.7)	29 (12.8)	
Working/retired	309 (66.2)	136 (55.1)	173 (78.6)	
**Education**	** **	** **	** **	**<0** **.** **0001**
High School or less	221 (47.2)	146 (59.1)	75 (33.9)	
Higher education	247 (52.8)	101 (40.9)	146 (66.1)	
**ADI National**	66.0 ± 22.9	74.9 ± 19.0	56.0 ± 22.8	**<** **.** **0001**
**SVI Census**	7.6 ± 1.9	8.4 ± 1.8	6.8 ± 1.7	**<** **.** **0001**
**Current Smoker, *N* (%)**	106 (22.9)	78 (32.1)	28 (12.7)	**<** **.** **0001**
**Insurance, *N* (%)**	215 (45.9)	121 (49.0)	94 (42.5)	0.1626
**Life Orientation Test-Revised, M ± SD**	17.6 ± 4.6	17.4 ± 4.3	17.9 ± 4.9	0.1075
**Experience of Discrimination, M ± SD**	7.2 ± 9.4	11.2 ± 10.3	2.8 ± 5.7	**<0** **.** **0001**
**Multidimensional Scale of Perceived Social Support, M ± SD**	65.4 ± 18.5	64.6 ± 19.4	66.2 ± 17.4	0.6099
**Perceived Stress Scale, M ± SD**	14.4 ± 6.3	14.9 ± 6.3	13.9 ± 6.3	**0** **.** **0477**
**Demographics/Covariates**				
**Age, M ± SD**	57.3 ± 7.7	55.8 ± 6.6	59.0 ± 8.4	**<0** **.** **0001**
**Gender, *N* (%)**				0.6053
Male	173 (37.0)	94 (38.1)	79 (35.7)	
Female	295 (63.0)	153 (61.9)	142 (64.3)	
**No. Comorbidities (0–14), *N* (%)**				0.0900
0	145 (31.0)	70 (28.3)	75 (34.0)	
1–2	249 (53.2)	136 (55.1)	113 (51.1)	
3+	74 (15.8)	41 (16.6)	33 (14.9)	
**No. Pain Sites**				0.1934
0–1	58 (12.4)	30 (12.1)	28 (12.7)	
2–3	109 (23.3)	53 (21.5)	56 (25.3)	
4+	301 (64.3)	164 (66.4)	137 (62.0)	
**Body Mass Index, M ± SD**	31.9 ± 7.7	33.0 ± 8.0	30.7 ± 7.1	**0** **.** **0014**
**Clinical Pain, Physical Function, and Experimental Pain**				
**Graded Chronic Pain Scale-Characteristic Pain Intensity, M ± SD**	53.2 ± 23.1	62.1 ± 22.4	43.2 ± 19.6	**<0** **.** **0001**
**Western Ontario and McMaster Universities Arthritis Index Pain, M ± SD**	7.6 ± 4.3	8.7 ± 4.4	6.4 ± 4.0	**<0** **.** **0001**
**Graded Chronic Pain Scale-Disability, M ± SD**	45.9 ± 29.7	53.2 ± 29.4	37.8 ± 27.9	**<0** **.** **0001**
**Western Ontario and McMaster Universities Arthritis Index Physical Function, M ± SD**	24.5 ± 14.9	28.9 ± 15.0	19.5 ± 13.2	**<0** **.** **0001**
**Temporal Summation- Knee, M ± SD**	18.7 ± 18.1	23.3 ± 18.5	13.5 ± 16.1	**<0** **.** **0001**
**Temporal Summation- Hand, M ± SD**	14.3 ± 17.0	19.8 ± 18.5	8.3 ± 12.8	**<0** **.** **0001**

NHB, non-hispanic black; NHW, non-hispanic white.

### 4.2. Clinical pain

Combined, environmental and behavioral factors explained 22.3% of the GCPS CPI and 16.2% of the WOMAC pain (Table 2). Covariates accounted for an additional 8.0% of the GCPS CPI and 9.3% of the WOMAC pain. Sociodemographic group accounted for an additional 5.0% variance of the GCPS CPI and 1.0% of the WOMAC pain. In the final model, lower income, social vulnerability index, perceived social support, sociodemographic resources, greater number of pain sites and BMI were significantly associated with higher GCPS CPI. Lower income, employment status, social vulnerability index, sociodemographic resources, greater number of pain sites and BMI were significantly associated with higher WOMAC pain.

**Table 2:** Nested linear regression models assessing environmental and behavioral factors on clinical pain.

	GCSP Pain intensity	WOMAC Pain
**MODEL 1**		
Income	**−0** **.** **1781** **	**−0** **.** **2267** **
Employment	−0.0613	−0.0166
Education	**−0** **.** **1782** **	**−0** **.** **1306** *
ADI National	0.0828	0.0035
SVI Census	**−0** **.** **1045** *	−0.0867
Smoking	−0.0048	0.0116
LOT-R	−0.0351	−0.0433
EOD	**0** **.** **1926** ***	**0** **.** **1943** ***
MSPSS	**−0** **.** **0976** *	−0.0237
PSS	0.0665	0.072
*Adj. R^2^*	0.2225	0.1622
**MODEL 2**		
Income	**−0** **.** **1892** **	**−0** **.** **2255** **
Employment	−0.0304	0.0015
Education	**−0** **.** **1256** *	−0.0687
ADI National	0.0831	0.0130
SVI Census	**−0** **.** **1207** *	−0.1032
Smoking	0.0256	0.0453
LOT-R	−0.0234	−0.0236
EOD	**0** **.** **1413** **	**0** **.** **1431** **
MSPSS	**−0** **.** **092** *	−0.0132
PSS	0.0328	0.0558
Age	**−0** **.** **1223** *	−0.06
Sex	−0.0354	−0.0525
No. Comorbidities	−0.0127	0.0391
No. Pain Sites	**0** **.** **2023** ***	**0** **.** **2457** ***
BMI	**0** **.** **1680** **	**0** **.** **1713** **
*Adj. R^2^*	0.3029	0.2550
Δ*R*^2^	0.0804	0.0928
**MODEL 3**		
Income	**−0** **.** **1408** *	**−0** **.** **2020** **
Employment	−0.0299	**0** **.** **0017** **
Education	−0.0998	−0.0564
ADI National	0.0603	0.0021
SVI Census	**−0** **.** **1807** *	**−0** **.** **1319** *
Smoking	0.0172	0.04138
LOT-R	−0.0450	−0.0342
EOD	0.0355	0.0924
MSPSS	**−0** **.** **0939** *	−0.0142
PSS	0.0470	0.0626
Age	−0.0921	−0.0452
Sex	−0.0464	−0.0579
No. Comorbidities	−0.0053	0.0425
No. Pain Sites	**0** **.** **2245** ***	**0** **.** **2565** ***
BMI	**0** **.** **1480** **	**0** **.** **1620** **
Sociodemographic Groups	**−0** **.** **2887** ***	**−0** **.** **1387** *
*Adj. R* ^2^	0.3527	0.2649
Δ*R*^2^	0.0498	0.0099

ADI, Area Deprivation Index; SVI, Social Vulnerability Index; PSS, Perceived Stress Scale; EOD, Experience of Discrimination; MSPSS, Multidimensional Scale of Perceived Social Support; BRS = Brief Resilience Scale; LOT-R, Life Orientation Test Revised.

Standardized (beta) values reported.

* *p* < 0.05.

** *p* < 0.01.

*** *p* < 0.0001.

### 4.3. Physical function

Environmental and behavioral factors explained 18.5% of the GCPS disability and 18.8% of the WOMAC function (Table 3). Covariates explained an additional 6.8% of the GCPS disability and 8.9% WOMAC function. Sociodemographic group accounted for an additional 1.4% variance in GCPS disability and 1.3% of variance in WOMAC function. In the final model, greater perceived stress, number of pain sites, BMI and lower sociodemographic resources were associated with higher GCPS disability. Lower income, social vulnerability index, sociodemographic resources, greater experience of discrimination, perceived stress, number of pain sites, and BMI were associated with worse WOMAC function.

**Table 3:** Nested linear regression models assessing environmental and behavioral factors on physical function.

	GCSP Disability	WOMAC Function
**MODEL 1**		
Income	−0.1142	**−0** **.** **1744** **
Employment	**−0** **.** **1181** *	−0.0124
Education	−0.0769	**−0** **.** **1247** *
ADI National	0.0118	0.065
SVI Census	−0.0311	−0.0896
Smoking	−0.0383	−0.0144
LOT-R	−0.0598	−0.0362
EOD	**0** **.** **1628** **	**0** **.** **2502** ***
MSPSS	−0.0001	−0.0182
PSS	**0** **.** **2200** ***	**0** **.** **1206** *
*Adj. R^2^*	0.1853	0.1883
**MODEL 2**		
Income	−0.1172	**−0** **.** **1628** *
Employment	−0.1034	−0.0047
Education	−0.0245	−0.0740
ADI National	0.0127	0.069
SVI Census	−0.0444	−0.1009
Smoking	−0.0046	0.0242
LOT-R	−0.0418	−0.014
EOD	**0** **.** **1125** *	**0** **.** **2038** ***
MSPSS	0.0068	−0.0118
PSS	**0** **.** **2022** **	**0** **.** **1177** *
Age	−0.0688	−0.0055
Sex	−0.0372	−0.05
No. Comorbidities	0.0565	0.0420
No. Pain Sites	**0** **.** **1708** **	**0** **.** **2209** ***
BMI	**0** **.** **1802** **	**0** **.** **2024** ***
*Adj. R^2^*	0.2534	0.2775
Δ*R*^2^	0.0681	0.0892
**MODEL 3**		
Income	−0.0901	**−0** **.** **1366** *
Employment	−0.1031	−0.0044
Education	−0.0101	−0.0600
ADI National	−0.0000	0.0566
SVI Census	−0.0778	**−0** **.** **1333** *
Smoking	−0.0093	0.0196
LOT-R	−0.0539	−0.0257
EOD	0.0534	**0** **.** **1466** **
MSPSS	0.0057	−0.0128
PSS	**0** **.** **2101** **	**0** **.** **1253** *
Age	−0.0519	0.0109
Sex	−0.0434	−0.0559
No. Comorbidities	0.0607	0.0460
No. Pain Sites	**0** **.** **1832** ***	**0** **.** **2329** ***
BMI	**0** **.** **1690** **	**0** **.** **1916** ***
Sociodemographic Groups	**−0** **.** **1611** **	**−0** **.** **1562** **
*Adj. R^2^*	0.2674	0.2906
Δ*R*^2^	0.0140	0.0131

ADI, Area Deprivation Index; SVI, Social Vulnerability Index; PSS, Perceived Stress Scale; EOD, Experience of Discrimination; MSPSS, Multidimensional Scale of Perceived Social Support; BRS = Brief Resilience Scale; LOT-R, Life Orientation Test Revised.

Standardized (beta) values reported.

* *p* < 0.05.

** *p* < 0.01.

*** *p* < 0.0001.

### 4.4. Experimental pain

Environmental and behavioral factors explained 1.6% of the variance of punctate temporal summation (TS) at the knee and 3.2% of the variance at the hand (Table 4). Covariates explained an additional 3.6% of variance for TS at knee and 0.3% at the hand. Sociodemographic groups accounted for an additional 5.7% variance at the knee and 9.6% variance at the hand. In the final model, higher age, female sex and lower sociodemographic resources were associated with punctate TS at the knee. In the final model, only lower sociodemographic resources were associated with punctate TS at the hand.

**Table 4:** Nested linear regression models assessing environmental and behavioral factors on experimental pain.

	Punctate TS Knee	Punctate TS Hand
MODEL 1		
Income	**−0** **.** **1699** *	**−0** **.** **1703** *
Employment	0.0521	0.0332
Education	0.0736	0.0173
ADI National	0.0844	0.0241
SVI Census	−0.0289	−0.0058
Smoking	−0.0572	**−0** **.** **1193** *
LOT-R	0.0229	−0.0103
EOD	0.0960	**0** **.** **1278** *
MSPSS	0.0220	0.0464
PSS	0.0265	0.0661
*Adj. R^2^*	0.0162	0.0320
MODEL 2		
Income	−0.1404	**−0** **.** **1602** *
Employment	0.0015	0.0190
Education	0.0628	0.0144
ADI National	0.0830	0.0149
SVI Census	−0.0263	−0.0050
Smoking	−0.0033	−0.0860
LOT-R	−0.0127	−0.0266
EOD	**0** **.** **1321** *	**0** **.** **1321** *
MSPSS	0.0309	0.0451
PSS	0.0037	0.0448
Age	0.0999	0.0094
Sex	**0** **.** **1992** **	0.0864
No. Comorbidities	−0.0882	−0.0609
No. Pain Sites	0.0656	0.0323
BMI	0.0116	0.0706
*Adj. R^2^*	0.0522	0.0351
Δ*R*^2^	0.0360	0.0031
**MODEL 3**		
Income	−0.0883	−0.0953
Employment	0.0018	0.0206
Education	0.0914	0.0505
ADI National	0.0578	−0.0189
SVI Census	−0.0903	−0.0875
Smoking	−0.0110	−0.0954
LOT-R	−0.0362	−0.0583
EOD	0.0193	−0.0147
MSPSS	0.0284	0.0417
PSS	0.0178	0.0605
Age	**0** **.** **1293** *	0.0470
Sex	**0** **.** **1882** **	0.0733
No. Comorbidities	−0.0792	−0.0488
No. Pain Sites	0.0890	0.0613
BMI	−0.0096	0.0421
Sociodemographic Groups	**−0** **.** **3086** ***	**−0** **.** **3993** ***
*Adj. R^2^*	0.1089	0.1315
Δ*R*^2^	0.0567	0.0964

ADI, Area Deprivation Index; SVI, Social Vulnerability Index; PSS, Perceived Stress Scale; EOD, Experience of Discrimination; MSPSS, Multidimensional Scale of Perceived Social Support; BRS = Brief Resilience Scale; LOT-R, Life Orientation Test Revised.

Standardized (beta) values reported.

* *p* < 0.05.

** *p* < 0.01.

*** *p* < 0.0001.

## 5. Discussion

The purpose of our study was to investigate factors that may contribute to previously reported ethnic/race group differences in pain-related outcomes. Based on available measures in the UPLOAD and UPLOAD-2 studies, we evaluated the contributions of environmental and behavioral factors as defined by the NIA and NIMHD Health Disparities Research Frameworks specific to 1) clinical pain, 2) physical function, and 3) experimental pain in mid to older adults with knee pain with or at risk for OA. *The NHB and NHW participants differed significantly across numerous sociodemographic variables. As only subgroups from each ethnic/race group were represented, ethnic/race groups comparisons were not interpretable and the term sociodemographic groups is used.* Our findings indicate that the inclusion of environmental and behavioral factors improved the models and reduced the variance observed between sociodemographic groups and the outcome measures. Additionally, pain sites, BMI, and income were predictive across several models. The current study provides initial findings in the development of an improved understanding of the complex array of factors contributing to previously reported ethnic/race group differences, a critical first step in addressing health disparities (61).

### 5.1. Clinical pain

Although investigations of environmental and sociocultural influences on clinical pain are not new, the body of findings have not shifted the general perception of ethnic/race group differences. However, evidence is building frequently showing environmental and sociocultural factors account for previously reported ethnic/race group differences (62, 63). Limited by available measures, our findings align with the growing body of research showing that environmental and behavioral factors with inclusion of known covariates explained 22 to 26% of the variance observed in clinical pain with sociodemographic groups adding only an additional 1 to 5%. Surprisingly, although the ADI national and SVI census show a positive correlation (rho = 0.66) and patterns are consistent with other environmental measures, the SVI census had a negative and significant association with the GCPS CPI and both measures of WOMAC. Mirroring our observed patterns, a recent population study by Zajacova and colleagues (2022), working with 2010–2018 data from the *National Health Interview Survey,* investigated pain prevalence across primary U.S. race groups with consideration for demographic and socioeconomic factors. Among an array of findings, investigators found black adults experience more severe clinical pain and lower prevalence of less severe pains than white adults. However, after accounting for SES and other covariates, black adults experienced significantly lower levels of severe pain than white adults (64). A second cross-sectional study with the 2019 data from the *National Health Interview Survey* essentially replicated prior findings with lower educational attainment predictive of higher odds of chronic pain and lower odds of effective pain management (65). Thus, consideration of environmental and sociocultural factors is not only conveying a reduction in previously indicated ethnic/race group differences, but a pattern of findings is developing elucidating which factors are the greater contributors to pain related disparities.

### 5.2. Physical function

Many self-report pain questionnaires include physical function questions, thus physical function is frequently included as an outcome with clinical pain. Not surprisingly, our physical function findings were similar to those for clinical pain. Environmental and behavioral factors with inclusion of known covariates explained 25 to 26% of the variance observed in physical function with sociodemographic groups adding only an additional 1% of explanatory value. Performance based physical function with consideration for environmental and sociocultural factors is further informative. Clay and colleagues compared the Short Performance Physical Battery (SPPB) in older NHW and NHB men with consideration for sociodemographic factors. NHB men had significantly lower SPPB performance (66). Cognitive functioning was the strongest predictor of physical performance. The study was bolstered by a large sample size and approximately equal number of participants for both groups. However, the groups were not balanced on sociodemographic and health related factors with the NHB having the greatest sociodemographic and health related risk factors. *Thus, ethnicity/race differences need to be interpreted with caution*. A number of studies have evaluated sociodemographic factors, housing conditions, and physical function using the SPPB (67–69). Addressing the sociodemographic and environmental group disparities in prior studies, Thorpe and colleagues analyzed data from NHB and NHW adults with similar socioeconomic and community backgrounds. Findings indicated the NHW adults had lower physical function than NHB adults (70). Progress is underway toward disentangling the complex array of factors contributing to previously reported ethnic/race group physical function disparities.

### 5.3. Experimental pain

Previous research on ethnic/race group differences in experimental pain have mirrored clinical pain and physical functioning findings (10). In the current study, environmental and behavioral factors and covariates accounted for less of the variance in punctate TS with sociodemographic groups explaining <10% of the variance. Cautions are warranted regarding comparisons with the meta-analysis cited as the UPLOAD findings were included multiple times in some categories (10). Ethnic/race group differences in nociceptive sensitivity have also been reported in individuals without chronic pain (71–73). Losin and colleagues found however, the difference in sensitivity in pain free NHB compared to Hispanic and NHW adults was not in brain-related nociceptive areas but frontostriatal activation associated with pain rating, discrimination, trust, and other external factors (74). Relationships between experimental pain and marital status have been reported (75). Additionally, income satisfaction accounted for differences observed in morning waking cortisol and morning waking slope (40). Further, socioeconomic status influences brain functional networks and anatomy (76, 77). Thus, as socioenvironmental stress gets “under the skin,” life experiences and chronic stress alter physiological systems, including nociceptive sensitivity (78, 79).

### 5.4. Additional considerations

First, research moving forward should consider if ethnic/race groups are similar based on sociodemographic factors. When groups significantly differ on relevant sociodemographic resources, a complete representation of either group has not been obtained. Inclusion of the imbalanced variables as a covariate in modeling does not “balance” the groups. Thus, ethnic/race group interpretations would be misleading. Reporting findings with consideration for various sociodemographic variables aligns with the concept of “intersectionality” (80, 81). For example, a sample description might be older, lower sociodemographic resources, NHB group compared to a younger, greater sociodemographic resources, NHW group. Such specificity will improve interpretability of findings and comparisons across studies. Second, studies with larger sample sizes of underrepresented groups are needed (7). Third, much of the existing research investigating differences in pain experiences between ethnic/race groups has focused on identifying and examining behavioral factors, with fewer studies evaluating environmental and sociocultural factors and the *combination* of factors across different levels of analysis (7). Fourth, two Health Disparities Research Frameworks have been developed (22, 23). Researchers, funders, and reviewers have the opportunity to combine efforts and promote the similarities between the two models, forging ahead together collaboratively to move the science forward expediently with the intention of reducing disparate outcomes in under-represented ethnic/race groups.

### 5.5. Strengths, limitations, and future directions

This study has many notable strengths. This study includes a large sample of community-dwelling mid to older adults with knee pain. Second, we measured clinical pain, physical function, and experimental pain. Third, the studies included different levels of analysis (environmental and behavioral) and different levels of influence (individual and ecological data). There are also limitations to acknowledge. First, environmental and behavioral factors were limited to the data available in the datasets. There may be other measures that better capture the factors addressed and extend beyond the measures included in this study. Additionally, future research, including investigations of the role of sociocultural factors are needed. Second, improved collection of environmental data are needed. Employment and insurance status measures were nominal in nature. Also, improved measures of income that take into consideration cumulative household finances and number of members would be more informative. Additionally, community/ecological variables are helpful however, we observed varied direction between the ADI and SVI measures and significance between the SVI and clinical pain and function in an unanticipated direction. Further research is needed to better understand how to incorporate community level measures in studies investigating health-related outcomes. Third, many of the questionnaires are specific to short time periods. Studies show that childhood stressors such as income inequality may have long lasting negative consequences for adult health (82). Finally, as the NHW and NHB participants differed significantly on multiple sociodemographic factors, interpretability and generalizability are limited.

## 6. Conclusion

There are a plethora of publications reporting ethnic/race group differences in pain-related experiences. To address the observed disparities, contributing factors need to be identified. Research across health conditions including pain show that with consideration for environmental and sociodemographic factors, ethnic/race differences are reduced or eliminated (62–65, 70, 83, 84). Our findings indicate a combined consideration of environmental and behavioral factors explained a significant proportion of variance in clinical pain, physical function and some of the variance for experimental pain. Although sociodemographic group differences remained, inclusion of the environmental and behavioral variables helped elucidate factors contributing to disparities in pain-related outcomes. An improved understanding of the complex array of factors contributing to health disparities is needed in advance of initiatives to improve health outcomes.

## Data Availability

The raw data supporting the conclusions of this article will be made available by the authors, without undue reservation.
